# Rare germline mutations in the *BRCA2* gene are associated with early-onset prostate cancer

**DOI:** 10.1038/sj.bjc.6603929

**Published:** 2007-08-14

**Authors:** I Agalliu, E Karlins, E M Kwon, L M Iwasaki, A Diamond, E A Ostrander, J L Stanford

**Affiliations:** 1 Division of Public Health Sciences, Fred Hutchinson Cancer Research Center, 1100 Fairview Avenue North, Seattle, WA 98109, USA; 2 Cancer Genetics Branch, National Human Genome Research Institute, National Institutes of Health, 50 South Drive, MSC 8000, Building 50, Bethesda, MD 20892, USA; 3 Edinburgh Molecular Genetics Service, Molecular Medicine Centre, Western General Hospital, Crewe Road, Edinburgh EH4 2XU, UK; 4 Department of Epidemiology, School of Public Health and Community Medicine, University of Washington, Mail Box 357236, Seattle, WA 98195, USA

**Keywords:** *BRCA2* gene, protein-truncating *BRCA2* mutations, prostate cancer, early-onset disease

## Abstract

Studies of families who segregate *BRCA2* mutations have found that men who carry disease-associated mutations have an increased risk of prostate cancer, particularly early-onset disease. A study of sporadic prostate cancer in the UK reported a prevalence of 2.3% for protein-truncating *BRCA2* mutations among patients diagnosed at ages ⩽55 years, highlighting the potential importance of this gene in prostate cancer susceptibility. To examine the role of protein-truncating *BRCA2* mutations in relation to early-onset prostate cancer in a US population, 290 population-based patients from King County, Washington, diagnosed at ages <55 years were screened for germline *BRCA2* mutations. The coding regions, intron–exon boundaries, and potential regulatory elements of the *BRCA2* gene were sequenced. Two distinct protein-truncating *BRCA2* mutations were identified in exon 11 in two patients. Both cases were Caucasian, yielding a mutation prevalence of 0.78% (95% confidence interval (95%CI) 0.09–2.81%) and a relative risk (RR) of 7.8 (95%CI 1.8–9.4) for early-onset prostate cancer in white men carrying a protein-truncating *BRCA2* mutation. Results suggest that protein-truncating *BRCA2* mutations confer an elevated RR of early-onset prostate cancer. However, we estimate that <1% of early-onset prostate cancers in the general US Caucasian population can be attributed to these rare disease-associated *BRCA2* mutations.

Studies of families segregating *BRCA2* mutations (BCLC, 1999; Johannsson *et al*, 1999; Eerola *et al*, 2001; Tulinius *et al*, 2002; Bermejo and Hemminki, 2004; van Asperen *et al*, 2005), kin-cohort studies of cancer incidence among relatives of population-based breast or ovarian cancer cases (Loman *et al*, 2003; Risch *et al*, 2006), as well as studies of populations who harbour founder *BRCA2* mutations (Sigurdsson *et al*, 1997; Struewing *et al*, 1997, Kirchhoff *et al*, 2004) suggest that men who carry a disease-associated *BRCA2* allele have an increased relative risk (RR) of prostate cancer (two- to five-fold elevation). Two of the family studies in which data were stratified on age at diagnosis of prostate cancer reported that the RR was even higher (seven- to eight-fold increase) in men diagnosed before age 65 years (BCLC, 1999; van Asperen *et al*, 2005). These findings suggest that protein-truncating *BRCA2* mutations may play a role in prostate cancer susceptibility. However, the majority of studies that have investigated the role of *BRCA2* mutations in hereditary prostate cancer (HPC) families, which usually include men diagnosed with prostate cancer at younger ages, have reported no disease-associated mutations (Wilkens *et al*, 1999; Gayther *et al*, 2000; Sinclair *et al*, 2000; Agalliu *et al*, 2007).

To date, only one study has assessed the contribution of *BRCA2* mutations in early-onset sporadic prostate cancer (Edwards *et al*, 2003). In a case series of 263 men in the UK diagnosed with prostate cancer at younger ages (⩽55 years), and who were not selected on the basis of either breast or prostate cancer family history, the prevalence of protein-truncating *BRCA2* mutations was 2.3% (95% confidence interval (95%CI) 0.8–5.0%) (Edwards *et al*, 2003). The estimated RR of prostate cancer was 23.0 (95%CI 9.0–57.0) among protein-truncating *BRCA2* mutation carriers in that study. The main goal of the current study was to determine the frequency of germline protein-truncating *BRCA2* mutations in early-onset prostate cancer patients in a US population.

## MATERIALS AND METHODS

### Study population

Case patients (*n*=290) included in this analysis are Caucasian and African American men diagnosed with histologically confirmed adenocarcinoma of the prostate before age 55 years and who participated in one of two population-based case–control studies of prostate cancer conducted in King County, Washington. Case patients in the first study (*n*=168) were diagnosed from January 1, 1993 to December 31, 1996 (Stanford *et al*, 1999), and those in the second study (*n*=128) were diagnosed from January 1, 2002 to December 31, 2005. Incident cases in both studies were identified through the population-based Seattle-Puget Sound SEER cancer registry, which also provided information on Gleason score, tumour stage, and serum prostate-specific antigen (PSA) level at diagnosis. Case patients completed a structured in-person interview that collected information about demographic and lifestyle characteristics, medical history, prostate cancer screening history, and family history of prostate cancer and other cancers. After the interview, patients were asked to provide a blood sample.

A total of 429 eligible prostate cancer case patients aged <55 years were identified during the ascertainment periods. Of eligible patients, 381 (89%) were interviewed and 301 (79%) provided a blood sample. A total of 290 case patients with adequate amounts of DNA were sequenced for *BRCA2* mutations. The study was approved by the Institutional Review Board of the Fred Hutchinson Cancer Research Center and the National Human Genome Research Institute, and written informed consent was obtained from all study participants.

### *BRCA2* gene sequencing

Genomic DNA was purified from peripheral blood lymphocytes using a standard proteinase K digestion and alkaline lysis followed by phenol–chloroform extraction (Sambrook *et al*, 1989). All coding and non-coding regions of the *BRCA2* gene were screened for mutations using 47 primer pairs described previously (Malone *et al*, 2000, 2006; Agalliu *et al*, 2007). Exons 5 and 6 were amplified in a single fragment and longer exons were amplified using multiple PCR reactions. There were five amplicons for exon 10 (10A–10E), 16 for exon 11 (11A–11P), and two for each of the following exons: 14 (14A and 14B), 18 (18A and 18B), and 27 (27A and 27B). PCR reactions were performed in a 10 *μ*l volume containing 5 ng of genomic DNA, 1 mM dNTPs, 1.5 mM, or 2.5 mM MgCl_2_, 1 *μ*M of both the forward and reverse primers, and 0.25 U Amplitaq DNA polymerase (Applied Biosystems, Foster City, CA, USA).

PCR products were treated using the ExoSAP-IT method (USB Corporation, Cleveland, OH, USA). DNA-sequencing reactions were carried out using BigDye® Terminator v3.1 Cycle Sequencing Kits (Applied Biosystems, Foster City, CA, USA) and sequence data were obtained on both forward and reverse PCR primers using the ABI 3730xl Genetic Analyzer (Applied Biosystems, Foster City, CA, USA). Contigs were assembled using PhredPhrap/Consed (Gordon *et al*, 1998). Variants were detected using Polyphred (Nickerson *et al*, 1997) and MutationSurveyor (http://www.softgenetics.com/mutationSurveyor.html) by two independent researchers. All variants were unanimously confirmed.

### Statistical analyses

The 95% CI for the prevalence of protein-truncating *BRCA2* mutations among prostate cancer patients was computed using a Poisson-distributed events-table under the assumption that these mutations are rare and follow a Poisson distribution. The prevalence of germline protein-truncating *BRCA2* mutations in the general population was taken from a recently published study of US women aged 35–64 years (Malone *et al*, 2006), and was used to estimate the RR of prostate cancer in men aged <55 years associated with carrying a *BRCA2* mutation.

A *χ*^2^ test was used to evaluate whether the distribution of genotypes for single nucleotide polymorphisms (SNPs) in the *BRCA2* gene differed by race (Caucasians *vs* African Americans), family history of prostate cancer (yes *vs* no) or clinical characteristics such as Gleason score (2–6 or 7=3+4 *vs* 7=4+3 or 8–10), stage of cancer (localised *vs* regional or distant) or a composite measure of disease aggressiveness. The definition of more aggressive prostate cancer included a Gleason score of 7=4+3 or 8–10 or regional or distant stage or serum PSA ⩾20 ng ml^−1^ at prostate cancer diagnosis; men with a Gleason score of 2–6 or 7=3+4 and local stage tumours and a serum diagnostic PSA level <20 ng ml^−1^ at diagnosis were classified as having less aggressive cancer. SAS version 9.1 (SAS Institute, Cary, NC, USA) was used for statistical analyses.

## RESULTS

Table 1 shows characteristics of the 290 population-based prostate cancer patients screened for *BRCA2* mutations; 11% were African-American and 2% were Jewish. The proportion of patients that reported either a first- or second-degree family history of prostate, breast, or ovarian cancer was 37, 16, and 3%, respectively. Five percent reported a family history of prostate, breast, and/or ovarian cancers; 3% reported first-degree relatives with either prostate or breast cancers. Most of the patients (79%) reported that they had received at least one PSA test and/or digital rectal examination (DRE) in the 5-year period before diagnosis. The majority of the patients had localised tumours (73%), Gleason scores of 2–6 (59%), and 36% of them were classified as having tumours with more aggressive clinical features at diagnosis.

Two distinct protein-truncating *BRCA2* mutations were identified, yielding an overall prevalence of 0.69% (95%CI 0.08–2.49%). Table 2 shows characteristics of the two prostate cancer patients with protein-truncating *BRCA2* mutations. One of these mutations was a 5-bp deletion (4625_4629delACATT) and the other was a 2-bp deletion (4074_4075delGT), both located in exon 11. Both cases with protein-truncating *BRCA2* mutations were Caucasian, one reported a first-degree relative with prostate cancer, but neither reported a family history of breast or ovarian cancer, and both were diagnosed with Gleason score 7 tumours. As the protein-truncating *BRCA2* mutations were detected in Caucasians only, the prevalence of such mutations in whites is 0.78% (95%CI 0.09–2.81%). Recently, Malone *et al* (2006) reported a weighted prevalence of protein-truncating *BRCA2* mutations of 0.1% (95%CI 0.0–0.3%) among US population-based Caucasian control women aged 35–64 years. Assuming that the prevalence of disease-associated *BRCA2* mutations among Caucasian men in the general population is similar to the prevalence of such mutations reported for US Caucasian women, the estimated RR of prostate cancer in white men <55 years of age associated with carrying a protein-truncating *BRCA2* mutation is 7.78 (95%CI 1.80–9.37).

In addition to the two protein-truncating mutations, 84 sequence variants were detected in the *BRCA2*-coding exons, intron–exon boundaries, and putative regulatory regions that were sequenced. One SNP was in the 5′ UTR region, 19 (22.6%) were intronic SNPs, and 40 (47.6%) non-synonomous and 24 (28.6%) synonomous SNPs were in coding exons. Most of these 84 SNPs were rare; only 11 (13.1%) had a minor allele frequency of 3% or greater, and of these, only four resulted in an amino-acid change (Table 3). There were two SNPs that differed (*P*<0.05) between Caucasian and African-American patients; both the C allele of SNP 1341A>C located in exon 10 and the T allele of the intronic SNP (IVS17-14) were more common in white than African American patients (26.7 *vs* 7.6% and 48.8 *vs* 34.8%, respectively). The distribution of genotypes for the SNPs having a minor allele frequency of ⩾3% did not vary substantially according to family history of prostate cancer or clinical features of the disease (data not shown). One exception was that the frequency of the T allele for the SNP 5971C>T located in exon 11 was higher in patients with more aggressive disease in comparison to cases with less aggressive clinical features, 5.2 *vs* 1.9%, respectively (*P*=0.02).

## DISCUSSION

In this population-based study of 290 patients diagnosed with prostate cancer before age 55 years, there were two patients with germline protein-truncating *BRCA2* mutations, yielding an overall prevalence of 0.69% (95%CI 0.08–2.49%). One of the cases with a *BRCA2* mutation reported a first-degree relative with prostate cancer, but neither reported a family history of breast or ovarian cancer. As both disease-associated *BRCA2* mutation carriers were Caucasian, the prevalence of such mutations in whites is 0.78% (95%CI 0.09–2.81%); the estimated RR of prostate cancer is 7.8 (95%CI 1.8–9.4) in Caucasian *BRCA2* mutation carriers. Although this represents a substantially elevated RR of earlier-onset prostate cancer associated with carrying a germline protein-truncating *BRCA2* mutation, such mutations are very rare (∼0.1%) in the general US population (Malone *et al*, 2006). The estimated cumulative risk (i.e. penetrance) of prostate cancer to age 55 years in Caucasian men due to carrying a disease-associated *BRCA2* mutation is about 1.1%, and the population attributable risk of prostate cancer in this same group is 0.7%.

In a hospital-based series (*n*=263) of prostate cancer patients with early ages at diagnosis (⩽55 years) in the UK, Edwards *et al* (2003) reported a prevalence of germline protein-truncating *BRCA2* mutations of 2.3% (95%CI 0.8–5.0%). The estimated RR of developing prostate cancer by age 56 years from a deleterious germline *BRCA2* mutation was 23.0 (95%CI 9.0–57.0). To compute this RR estimate, Edwards *et al* (2003) used the average of the estimated prevalence (i.e., 0.12 and 0.07%) of germline disease-associated *BRCA2* mutations in the general UK population, which was indirectly estimated from two prior studies of breast cancer in the UK (Peto *et al*, 1999; Antoniou *et al*, 2002). Interestingly, of the six prostate cancer patients carrying disease-associated *BRCA2* mutations, 50% were diagnosed before age 50 and most had no family history of prostate cancer (five out of six) or breast cancer (four out of six) (Edwards *et al*, 2003).

Although the overall finding is consistent with our results, a lower prevalence of disease-associated *BRCA2* mutations was observed in our population-based patient series. This difference may be due to different study designs and populations. Edwards *et al* (2003) utilised prostate cancer patients recruited through three different sources: Cancer Research UK/British Prostate Group, UK Familial Prostate Cancer Study, and the British Association of Urological Section of Oncology, and 90% of them had clinically detected prostate cancer. By contrast, our patients were ascertained from a population-based SEER registry. Most of these men had received a PSA and/or DRE test within the 5-year period before diagnosis, and 27% of the cases reported one or more clinical signs or symptoms at the time of diagnosis.

Studies of families with breast and/or ovarian cancer who harbour disease-associated *BRCA2* mutations have reported that male family members who carry such mutations have an increased RR of prostate cancer (BCLC, 1999; Johannsson *et al*, 1999; Eerola *et al*, 2001; Tulinius *et al*, 2002; Bermejo and Hemminki, 2004; van Asperen *et al*, 2005). In the Breast Cancer Linkage Consortium cohort of 173 breast and/or ovarian cancer families, a RR of 4.7 (95%CI 3.5–6.2) was reported for prostate cancer among *BRCA2* protein-truncating mutation carriers (BCLC, 1999). In that study, the RR was higher in men diagnosed at ages <65 years (RR=7.3, 95%CI 4.7–11.5). A Finnish study (Eerola *et al*, 2001) of 107 breast or ovarian cancer families reported a five-fold increase in the RR of prostate cancer among men carrying protein-truncating *BRCA2* mutations (RR=4.9, 95%CI 1.8–11.0). Similar findings were reported by Tulinius *et al* (2002) in an analysis of 995 breast cancer pedigrees from Iceland. In that study, first-degree male relatives of breast cancer patients carrying protein-truncating *BRCA2* mutations had a RR of 4.8 (95%CI 3.3–6.3) for prostate cancer (Tulinius *et al*, 2002). Finally, van Asperen *et al* (2005) in a analysis of 139 *BRCA2* families from the Netherlands reported an RR of 2.5 (95%CI 1.6–3.8) for prostate cancer among *BRCA2* protein-truncating mutation carriers. When the analysis was stratified by the age at diagnosis, the RR of prostate cancer was 8.0 (95%CI 4.1–14.0) among protein-truncating *BRCA2* mutation carriers diagnosed at ages <65 years.

In contrast to the above studies and the current analysis, most studies of HPC families investigating the contribution of protein-truncating *BRCA2* mutations to prostate cancer susceptibility have reported null results (Wilkens *et al*, 1999; Gayther *et al*, 2000; Sinclair *et al*, 2000; Agalliu *et al*, 2007). Only one study conducted in the UK of 38 affected individuals from HPC families with two to five affected men per family reported a prevalence of 5.3% for protein-truncating *BRCA2* mutations (Gayther *et al*, 2000). In that study, the two men with protein-truncating *BRCA2* mutations were diagnosed with prostate cancer at ages ⩽56 years, and both had a brother with prostate cancer. There is still uncertainty as to why *BRCA2* families, ascertained on the basis of members with breast and/or ovarian cancer, have a higher RR of prostate cancer in men carrying protein-truncating *BRCA2* mutation (BCLC, 1999; Johannsson *et al*, 1999; Eerola *et al*, 2001; Tulinius *et al*, 2002; Bermejo and Hemminki, 2004; van Asperen *et al*, 2005). By comparison, when HPC families that include members with a history of breast or ovarian cancer are screened for *BRCA2* mutations, no excess of protein-truncating *BRCA2* mutations is found (Agalliu *et al*, 2007).

A more complete understanding of the role of the *BRCA2* gene in relationship with prostate cancer susceptibility may provide insight on prostate carcinogenesis. For example, the encoded BRCA2 protein is involved in DNA repair, transcription, and cell cycle control (Davies *et al*, 2001; Venkitaraman, 2002; Powell *et al*, 2003; Yoshida and Miki, 2004). BRCA2 interacts directly with RAD51, which is an essential protein for error-free double-strand DNA break repair by homologous recombination (Sharan *et al*, 1997; Wong *et al*, 1997; Xia *et al*, 2001; Pellegrini *et al*, 2002; Venkitaraman, 2002; Jackson, 2002; Powell *et al*, 2003). *BRCA2* regulates both the intracellular localisation and DNA-binding ability of RAD51, and therefore loss of this control following BRCA2 inactivation may be a key event leading to genomic instability and carcinogenesis (Davies *et al*, 2001; Jackson, 2002; Yoshida and Miki, 2004). Whether prostate tissue is particularly susceptible to *BRCA2* loss is not clear. The fact that protein-truncating mutations in the *BRCA2* gene predispose to breast and ovarian cancers suggests that the hormonal nature of these cancers may be important. In relation to the prostate, a study found that BRCA2 functions as a co-activator for the androgen receptor (AR) in conjunction with histone acetyltransferase (Shin and Verma, 2003). This finding suggests a novel mechanism by which BRCA2 protein may exert its tumour suppressor function, by altering AR signalling.

Our study has strengths and limitations that should be considered when interpreting the results. The current study is population-based; patients were not selected on the basis of family history of prostate, breast, or ovarian cancer, which offers a less-biased assessment of the role of *BRCA2* protein-truncating mutations in men from the general population. In addition, this is one of the largest such studies completed to date. A limitation of this study is that no screening for large genomic deletions in the *BRCA2* gene was performed, as this method requires a relatively large amount of DNA per patient. It has been estimated that large genomic deletions account for an additional 10–15% of reported disease-associated *BRCA2* mutations (Szabo and King, 1997). Thus, the prevalence of protein-truncating *BRCA2* mutations might have been underestimated in this study. In addition, owing to the high cost of genetic sequencing and the rarity of germline *BRCA2* protein-truncating mutations in the general population, it was not feasible to screen the large number of control men that would be required for direct case–control comparisons. Therefore, we used the previously reported prevalence of *BRCA2* mutations in Caucasian control women from the general US population (Malone *et al*, 2006) to estimate the RR. There is no reason to believe that the prevalence of *BRCA2* mutations in the general population would be substantially different between men and women, as the gene is located on chromosome 13. Malone *et al* (2006) reported a prevalence of 0.1% for germline *BRCA2* protein-truncating mutations among US Caucasian control women. This prevalence is similar to the estimates of 0.12 and 0.07% for *BRCA2* protein-truncating mutations in the general UK population (Peto *et al*, 1999; Antoniou *et al*, 2002). By contrast, a recent population-based study of ovarian cancer in Ontario, Canada, estimated a higher carrier frequency of protein-truncating *BRCA2* mutations of 0.69% (95%CI 0.43%–1.10%) in the general population (Risch *et al*, 2006). However, the Canadian estimate was based on data from ovarian cancer patients. In addition, the ovarian cancer patient population was enriched with subjects, such as Ashkenazi Jews, known to have a higher prevalence of *BRCA2* disease-associated founder mutations (Risch *et al*, 2006).

In conclusion, the results of this study indicate that germline protein-truncating mutations in the *BRCA2* gene confer an elevated RR (almost eight-fold) of developing early-onset prostate cancer in Caucasian men. However, the population attributable risk of prostate cancer in US Caucasians under age 55 due to carrying one of these rare germline protein-truncating *BRCA2* mutation is estimated to be <1%. Given the relatively high cost of *BRCA2* mutation screening and the rarity of these high-risk *BRCA2* protein-truncating mutations in the general population, screening for these mutations in the general population would not be cost effective and is not currently recommended.

## Figures and Tables

**Table 1:** Characteristics of 290 population-based prostate cancer patients aged <55 years at diagnosis screened for *BRCA2* mutations

	**1993–1996 (*n*=162)**		**2002–2005 (*n*=128)**		**Total case patients (*n*=290)**	
**Ascertainment period**	**N**	**%**	**N**	**%**	**N**	**%**
*Age at diagnosis (years)*						
35–49	39	24.1	58	45.3	97	33.4
50–54	123	75.9	70	54.7	193	66.6
						
*Race*						
Caucasian	154	95.1	103	80.5	257	88.6
African-American	8	4.9	25	19.5	33	11.4
						
*Jewish*						
No	159	98.1	126	98.4	285	98.3
Yes	3	1.9	2	1.6	5	1.7
						
*Family history of prostate cancer*						
None	111	68.5	71	55.5	182	62.8
First-degree	33	20.4	40	31.3	73	25.2
Second-degree only	18	11.1	17	13.3	35	12.1
						
*Family history of breast cancer*						
None	134	82.7	109	85.2	243	83.8
First-degree	22	13.6	15	11.7	37	12.8
Second-degree only	6	3.7	4	3.1	10	3.4
						
*Family history of ovarian cancer*						
None	157	96.9	123	96.1	280	96.6
First-degree	2	1.2	3	2.3	5	1.7
Second-degree only	3	1.9	2	1.6	5	1.7
						
Prostate cancer screening^a^						
None	32	19.8	28	21.9	60	20.7
DRE only	46	28.4	25	19.5	71	24.5
PSA and DRE	84	51.9	75	58.6	159	54.8
						
*Gleason score*						
2–4	19	11.7	—	—	19	6.6
5–6	82	50.6	69	53.9	151	52.1
7 (3+4)	39	24.1	40	31.3	79	27.2
7 (4+3)	9	5.6	11	8.6	20	6.9
8–10	10	6.2	6	4.7	16	5.5
Missing	3	1.9	2	1.6	5	1.7
						
*Stage of cancer*						
Localised	114	70.4	97	75.8	211	72.8
Regional	40	24.7	29	22.7	69	23.8
Distant	8	4.9	2	1.6	10	3.4
						
*PSA at diagnosis (ng/ml)* b						
0–3.9	26	16.1	29	22.7	55	19.0
4.0–9.9	77	47.6	63	49.2	140	48.3
10.0–19.9	21	12.9	15	11.7	36	12.4
⩾20	19	11.7	11	8.6	30	10.3
Missing	19	11.7	10	7.8	29	10.0

a One or more prostate-specific antigen (PSA) test or digital rectal exam (DRE) in the 5-year period before diagnosis date.

b Serum prostate-specific antigen (PSA) level at diagnosis.

**Table 2:** Characteristics of prostate cancer patients with germline protein-truncating *BRCA2* mutations

	**Case A**	**Case B**
*BRCA2 mutation*		
Exon location	11	11
Nucleotide change	4625_4629delACATT	4074_4075delGT
Protein effect	STOP 1467	STOP 1284
		
*Characteristics*		
Age at diagnosis (years)	51	47
Race	Caucasian	Caucasian
		
*Family history of*		
Prostate cancer	Yes (first-degree)	No
Breast cancer	No	No
Ovarian cancer	No	No
		
*Clinical characteristics*		
Gleason score	7 (3+4)	7 (4+3)
Stage of cancer	Localised	Localised
PSA at diagnosis (ng/ml)	Missing	6.3

**Table 3:** SNPs with a ⩾3% minor allele frequency in the *BRCA2* gene among 290 prostate cancer patients, by race

**Exon**	**Nucleotide position**	**Nucleotide change**	**Amino-acid change**	**Genotype**	**Total *N*=290**	**%**	**Caucasian *N*=257**	**%**	**African American *N*=33**	**%**	***P*-value^a^**
*SNP in the* *5′* *UTR region*											
	202	G>A	5′ UTR	GG	160	55.2	139	54.1	21	63.6	0.51
				AG	100	34.5	90	35.0	10	30.3	
				AA	30	10.3	28	10.9	2	6.1	
											
*Non-synonomous SNPs*											
10	1092	A>C	N289H	AA	269	92.8	240	93.4	29	87.9	0.25
				AC^b^	21	7.2	17	6.6	4	12.1	
10	1341	A>C	N372H	AA	170	58.6	142	55.3	28	84.8	0.004
				AC	98	33.8	93	36.2	5	15.2	
				CC	22	7.6	22	8.6	—	—	
11	3198	A>G	N991D	AA	268	92.4	240	93.4	28	84.8	0.08
				AG^b^	22	7.6	17	6.6	5	15.2	
11	5971	C>T	T1915M	CC	272	93.8	243	94.6	29	87.9	0.13
				CT^b^	18	6.2	14	5.4	4	12.1	
											
*Synonomous or intronic SNPs*											
10	1592	A>G	S455S	AA	269	92.8	240	93.4	29	87.9	0.25
				AG^b^	21	7.2	17	6.6	4	12.1	
11	2456	T>C	H743H	TT	269	92.8	240	93.4	29	87.9	0.25
				CT^b^	21	7.2	17	6.6	4	12.1	
11	3623	A>G	K1132K	AA	134	46.2	121	47.1	13	39.4	0.68
				AG	121	41.7	105	40.9	16	48.5	
				GG	35	12.1	31	12.1	4	12.1	
11	4034	T>C	V1269V^c^	TT	195	67.2	171	66.5	24	72.7	0.72
				CT	77	26.6	69	26.8	8	24.2	
				CC	16	5.5	15	5.8	1	3.0	
14	7469	A>G	S2414S	AA	170	58.6	155	60.3	15	45.5	0.23
				AG	104	35.9	89	34.6	15	45.5	
				GG	16	5.5	13	5.1	3	9.1	
17	IVS17-14	T>C	intronic	CC	86	29.7	70	27.2	16	48.5	0.04
				CT	134	46.2	123	47.9	11	33.3	
				TT	70	24.1	64	24.9	6	18.2	

a *χ*^2^ *P*-value comparing the distribution of genotypes between Caucasian and African-American prostate cancer cases.

b No homozygous variants were detected.

c Two Caucasian men had missing data for this SNP and thus percentages do not add up to 100%.

## References

[bib1] Agalliu I, Kwon EM, Zadory D, McIntosh L, Thompson J, Stanford JL, Ostrander EA (2007) Germline mutations in the BRCA2 gene and susceptibility to hereditary prostate cancer. Clin Cancer Res 13: 839–843 17289875 10.1158/1078-0432.CCR-06-2164

[bib2] Antoniou AC, Pharoah PD, McMullan G, Day NE, Stratton MR, Peto J, Ponder BJ, Easton DF (2002) A comprehensive model for familial breast cancer incorporating BRCA1, BRCA2 and other genes. Br J Cancer 86: 76–83 11857015 10.1038/sj.bjc.6600008 PMC2746531

[bib3] Bermejo JL, Hemminki K (2004) Risk of cancer at sites other than the breast in Swedish families eligible for *BRCA1* or *BRCA2* mutation testing. Ann Oncol 15: 1834–1841 15550590 10.1093/annonc/mdh474

[bib4] Breast Cancer Linkage Consortium (BCLC) (1999) Cancer risks in BRCA2 mutation carriers. J Natl Cancer Inst 91: 1310–1316 10433620 10.1093/jnci/91.15.1310

[bib5] Davies AA, Masson JY, McIlwraith MJ, Stasiak AZ, Stasiak A, Venkitaraman AR, West SC (2001) Role of BRCA2 in control of the RAD51 recombination and DNA repair protein. Mol Cell 7: 273–282 11239456 10.1016/s1097-2765(01)00175-7

[bib6] Edwards SM, Kote-Jarai Z, Meitz J, Hamoudi R, Hope Q, Osin P, Jackson R, Southgate C, Singh R, Falconer A, Dearnaley DP, Ardern-Jones A, Murkin A, Dowe A, Kelly J, Williams S, Oram R, Stevens M, Teare DM, Ponder BA, Gayther SA, Easton DF, Eeles RA (2003) Two percent of men with early-onset prostate cancer harbor germline mutations in the BRCA2 gene. Am J Hum Genet 72: 1–12 12474142 10.1086/345310 PMC420008

[bib7] Eerola H, Pukkala E, Pyrhonen S, Blomqvist C, Sankila R, Nevanlinna H (2001) Risk of cancer in *BRCA1* and *BRCA2* mutation-positive and -negative breast cancer families (Finland). Cancer Causes Control 12: 739–746 11562114 10.1023/a:1011272919982

[bib8] Gayther SA, de Foy KA, Harrington P, Pharoah P, Dunsmuir WD, Edwards SM, Gillett C, Ardern-Jones A, Dearnaley DP, Easton DF, Ford D, Shearer RJ, Kirby RS, Dowe AL, Kelly J, Stratton MR, Ponder BA, Barnes D, Eeles RA (2000) The frequency of germline mutations in the breast cancer predisposition genes *BRCA1* and *BRCA2* in familial prostate cancer. Cancer Res 60: 4513–4518 10969800

[bib9] Gordon D, Abajian C, Green P (1998) Consed: A Graphical Tool for Sequence Finishing. Genome Res 8: 195–202 9521923 10.1101/gr.8.3.195

[bib10] Jackson SP (2002) Sensing and repairing DNA double-strand breaks. Carcinogenesis 23: 687–696 12016139 10.1093/carcin/23.5.687

[bib11] Johannsson O, Loman N, Moller T, Kristoffersson U, Borg A, Olsson H (1999) Incidence of malignant tumours in relatives of *BRCA1* and *BRCA2* germline mutation carriers. Eur J Cancer 35: 1248–1257 10615237 10.1016/s0959-8049(99)00135-5

[bib12] Kirchhoff T, Kauff ND, Mitra N, Nafa K, Huang H, Palmer C, Gulati T, Wadsworth E, Donat S, Robson ME, Ellis NA, Offit K (2004) BRCA mutations and risk of prostate cancer in Ashkenazi Jews. Clin Cancer Res 10: 2918–2921 15131025 10.1158/1078-0432.ccr-03-0604

[bib13] Loman N, Bladstrom A, Johannsson O, Borg A, Olsson H (2003) Cancer incidence in relatives of a population-based set of cases of early-onset breast cancer with a known *BRCA1* and *BRCA2* mutation status. Breast Cancer Res 5: R175–R186 14580253 10.1186/bcr632 PMC314401

[bib14] Malone KE, Daling JR, Doody DR, Hsu L, Bernstein L, Coates RJ, Marchbanks PA, Simon MS, McDonald JA, Norman SA, Strom BL, Burkman RT, Ursin G, Deapen D, Weiss LK, Folger S, Madeoy JJ, Friedrichsen DM, Suter NM, Humphrey MC, Spirtas R, Ostrander EA (2006) Prevalence and predictors of BRCA1 and BRCA2 mutations in a population-based study of breast cancer in white and black american women ages 35–64 years. Cancer Res 66: 8297–8308 16912212 10.1158/0008-5472.CAN-06-0503

[bib15] Malone KE, Daling JR, Neal C, Suter NM, O'Brien C, Cushing-Haugen K, Jonasdottir TJ, Thompson JD, Ostrander EA (2000) Frequency of BRCA1/BRCA2 mutations in a population-based sample of young breast carcinoma cases. Cancer 88: 1393–1402 10717622 10.1002/(sici)1097-0142(20000315)88:6<1393::aid-cncr17>3.0.co;2-p

[bib16] Nickerson DA, Tobe VO, Taylor SL (1997) PolyPhred: automating the detection and genotyping of single nucleotide substitutions using fluorescence-based resequencing. Nucleic Acids Res 25: 2745–2751 9207020 10.1093/nar/25.14.2745 PMC146817

[bib17] Pellegrini L, Yu DS, Lo T, Anand S, Lee M, Blundell TL, Venkitaraman AR (2002) Insights into DNA recombination from the structure of a RAD51-BRCA2 complex. Nature 420: 287–293 12442171 10.1038/nature01230

[bib18] Peto J, Collins N, Barfoot R, Seal S, Warren W, Rahman N, Easton DF, Evans C, Deacon J, Stratton MR (1999) Prevalence of BRCA1 and BRCA2 gene mutations in patients with early-onset breast cancer. J Natl Cancer Inst 91: 943–949 10359546 10.1093/jnci/91.11.943

[bib19] Powell SN, Kachnic LA (2003) Roles of BRCA1 and BRCA2 in homologous recombination, DNA replication fidelity and the cellular response to ionizing radiation. Oncogene 22: 5784–5791 12947386 10.1038/sj.onc.1206678

[bib20] Risch HA, McLaughlin JR, Cole DE, Rosen B, Bradley L, Fan I, Tang J, Li S, Zhang S, Shaw PA, Narod SA (2006) Population BRCA1 and BRCA2 mutation frequencies and cancer penetrances: a kin-cohort study in Ontario, Canada. J Natl Cancer Inst 98: 1694–1706 17148771 10.1093/jnci/djj465

[bib21] Sambrook J, Fritsch EF, Maniatis T (1989) Isolation of high-molecular weight DNA from mammalian cells. In Molecular Cloning: A Laboratory Manual Nolan C (ed), Vol 2, pp 9.16–9.19. Cold Spring Harbor Laboratory Press, New York

[bib22] Sharan SK, Morimatsu M, Albrecht U, Lim DS, Regel E, Dinh C, Sands A, Eichele G, Hasty P, Bradley A (1997) Embryonic lethality and radiation hypersensitivity mediated by Rad51 in mice lacking BRCA2. Nature 386: 804–810 9126738 10.1038/386804a0

[bib23] Shin S, Verma IM (2003) BRCA2 cooperates with histone acetyltransferases in androgen receptor-mediated transcription. Proc Natl Acad Sci USA 100: 7201–7206 12756300 10.1073/pnas.1132020100 PMC165853

[bib24] Sigurdsson S, Thorlacius S, Tomasson J, Tryggvadottir L, Benediktsdottir K, Eyfjord JE, Jonsson E. (1997) *BRCA2* mutation in Icelandic prostate cancer patients. J Mol Med 75: 758–761 9383000 10.1007/s001090050162

[bib25] Sinclair CS, Berry R, Schaid D, Thibodeau SN, Couch FJ (2000) *BRCA1* and *BRCA2* have a limited role in familial prostate cancer. Cancer Res 60: 1371–1375 10728701

[bib26] Stanford JL, Wicklund KG, McKnight B, Daling JR, Brawer MK (1999) Vasectomy and risk of prostate cancer. Cancer Epidemiol Biomarkers Prev 8: 881–886 10548316

[bib27] Struewing JP, Hartge P, Wacholder S, Baker SM, Berlin M, McAdams M, Timmerman MM, Brody LC, Tucker MA (1997) The risk of cancer associated with specific mutations of BRCA1 and BRCA2 among Ashkenazi Jews. N Engl J Med 336: 1401–1408 9145676 10.1056/NEJM199705153362001

[bib28] Szabo CI, King MC (1997) Population genetics of BRCA1 and BRCA2. Am J Hum Genet 60: 1013–1020 9150148 PMC1712447

[bib29] Tulinius H, Olafsdottir GH, Sigvaldason H, Arason A, Barkardottir RB, Egilsson V, Ogmundsdottir HM, Tryggvadottir L, Gudlaugsdottir S, Eyfjord JE (2002) The effect of a single *BRCA2* mutation on cancer in Iceland. J Med Genet 39: 457–462 12114473 10.1136/jmg.39.7.457 PMC1735175

[bib30] van Asperen CJ, Brohet RM, Meijers-Heijboer EJ, Hoogerbrugge N, Verhoef S, Vasen HF, Ausems MG, Menko FH, Gomez Garcia EB, Klijn JG, Hogervorst FB, van Houwelingen JC, van't Veer LJ, Rookus MA, van Leeuwen FE, Netherlands Collaborative Group on Hereditary Breast Cancer (2005) Cancer risks in BRCA2 families: estimates for sites other than breast and ovary. J Med Genet 42: 711–719 16141007 10.1136/jmg.2004.028829 PMC1736136

[bib31] Venkitaraman AR (2002) Cancer susceptibility and the functions of BRCA1 and BRCA2. Cell 108: 171–182 11832208 10.1016/s0092-8674(02)00615-3

[bib32] Wilkens EP, Freije D, Xu J, Nusskern DR, Suzuki H, Isaacs SD, Wiley K, Bujnovsky P, Meyers DA, Walsh PC, Isaacs WB (1999) No evidence for a role of *BRCA1* or *BRCA2* mutations in Ashkenazi Jewish families with hereditary prostate cancer. Prostate 39: 280–284 10344217 10.1002/(sici)1097-0045(19990601)39:4<280::aid-pros8>3.0.co;2-f

[bib33] Wong AKC, Pero R, Ormonde PA, Tavtigian SV, Bartel PL (1997) RAD51 interacts with the evolutionary conserved BRC motifs in the human cancer susceptibility gene BRCA2. J Biol Chem 272: 31941–31944 9405383 10.1074/jbc.272.51.31941

[bib34] Xia F, Taghian DG, DeFrank JS, Zeng ZC, Willers H, Iliakis G, Powell SN (2001) Deficiency of human BRCA2 leads to impaired homologous recombination but maintains normal nonhomologous end joining. Proc Natl Acad Sci 98: 8644–8649 11447276 10.1073/pnas.151253498 PMC37489

[bib35] Yoshida K, Miki Y (2004) Role of BRCA1 and BRCA2 as regulators of DNA repair, transcription, and cell cycle in response to DNA damage. Cancer Sci 95: 866–871 15546503 10.1111/j.1349-7006.2004.tb02195.x PMC11159131

